# Insurtech strategies: a comparison of incumbent insurance firms with new entrants

**DOI:** 10.1057/s41288-024-00341-0

**Published:** 2024-12-03

**Authors:** Christopher P. Holland, Anil S. Kavuri

**Affiliations:** 1 Loughborough Business School, Loughborough University, Leicester, LE11 3TL UK; 2 Alliance Manchester Business School, University of Manchester, Manchester, M15 6PB UK; 3 Münster University, Munster, Germany

**Keywords:** Insurtech, Business innovation, Artificial intelligence, Big data, New entrants, Incumbent firms

## Abstract

Insurtech is closely associated with digital transformation by new entrants that seek to disrupt insurance markets. However, the insurtech concept also includes its use by incumbent insurance companies, which are actively deploying a wide variety of insurtech applications to protect their market positions through innovation of their existing business models, e.g. through improved business processes or new insurance services. A theoretical insurtech business innovation model is developed that captures the effects of digital technology in insurance markets by considering innovation as a multi-dimensional concept that encompasses business processes, novel insurance products and changes to the insurance value chain. This framework is applied to an empirical sample of digital leaders: three incumbents and four new entrants. The results illustrate a variety of insurtech applications that include the transformation of business processes, products and new types of value chain configuration, as well as relatively minor enhancements to existing systems and business practices. It is shown that all the new entrants exploit artificial intelligence, big data and digital technology to build brand-new insurance services that emphasise innovative product features, high customer value and a delightful customer experience. In contrast, the legacy insurance firms tend to use digital technology in a defensive manner, e.g. the enhancement of existing insurance services, distribution channels and market positions. The exception is the launch of a telematics insurance service by an incumbent firm, where the telematics insurance effectively operates as a standalone business within a legacy insurance firm. The theory model is effective at analysing and evaluating both the type and magnitude of innovation. The case studies make an empirical contribution by illustrating state-of-the-art innovation by insurance disruptors and contrasts this with the defensive and sometimes novel digital strategies of incumbent firms. Future trends and research opportunities are outlined.

## Introduction

Our research analyses the emerging strategies from the deployment of insurtech and explores the differences between how new entrants and incumbent insurance firms adopt insurtech for the purpose of business transformation. It is often assumed in the business press and academe that insurtech is the sole province of new entrants that seek to disrupt the status quo of existing insurance markets. However, the picture is more complicated and nuanced because large incumbent firms are also using insurtech to redesign their business processes and develop innovative insurance services. To capture the strategies of both types of insurance firm, we develop and apply an innovation framework to a theoretical sample of leading incumbent insurance firms and new entrants.

The insurance market presents a timely opportunity to evaluate the competitive changes from digital disruption and business transformation. Insurance has arguably lagged other market sectors such as manufacturing, retail banking and e-commerce (Naylor 2017). Artificial intelligence (AI) is a crucial part of insurtech and is central to many of the recent innovations in insurance services (Behm et al. 2019; Balasubramanian et al. 2021a, b). This is reflected by significant investment in insurtech globally (Catlin et al. 2017), and a plethora of research reports by leading consultancy firms (Santenac et al. 2024; Balasubramanian et al. 2021a, b), which are testament to the expected benefits of AI in insurance markets. AI is creating a market discontinuity in many areas of insurance as incumbent firms transform legacy systems, new forms of data are used to assess and price risk, and radically different business models and technical methods are launched, facilitated by novel insurtech (Behm et al. 2019).

The structure of the paper is as follows: a brief review of topical insurtech literature is presented to justify (a) the development of a business innovation framework that captures the nature of innovation in insurance markets and the role of insurtech, and (b) the need for empirical research that compares and contrasts digital and AI leaders in the two groups of established incumbent insurance firms, and attackers. Conclusions are made regarding the contribution of the research and the managerial implications that stem from the expected trajectory of insurtech.

## Insurtech: incumbents and new entrants

Insurtech is a portmanteau that combines insurance with technology, though its meaning often varies when it is used by different authors and in a variety of market contexts (Balasubramanian et al. 2021a, b). The focus on different aspects of insurtech, such as its technical dimensions (Gorzata et al. 2021; Singh and Chivukula 2020), emphasis on AI technology (Zanke and Sontakke 2021) or new entrants and the disruptive capabilities of insurtech (Neale et al. 2020), are reflected in variations in the definition of insurtech (Gómez and Pineda 2023). The digital technology includes AI, digital infrastructure, big data analytics and other technologies such as sensors, GPS, natural language processing (NLP), robotic process automation (RPA) and, in the case of incumbents, legacy information systems.

Based on an extensive evaluation of the terminology used in the academic literature, the definition by Gomez and Pineda (2023) identifies the importance of technology and business factors. From a technology perspective, AI and big data are arguably the two most important digital phenomena in insurance (Buesnel et al. 2020; Holland and Kavuri 2021; European Commission 2020), and should therefore be considered as a system where they are interdependent to create a meaningful result. In an insurance context, the AI-big data system exists within a broader business process and information systems (IS) context. For example, an AI risk assessment application would exist within a risk assessment and management process and would draw data from a range of relevant IS such as legacy systems for historical claims data and policy information, and newer systems, e.g. GPS and new types of big data that can be used in insurance (EIOPA 2019). When AI is viewed in the context of an AI system that encapsulates big data and the business process or problem context, then it becomes clear that it is a ‘general purpose technology’ (GPT) (Trajtenberg 2019), i.e. it has the potential to transform a whole industry or market, rather than just a specific production technology or innovation.

This lack of agreed nomenclature is common in new and rapidly emerging technology markets. What is clear though is that insurtech must be considered within a broader market context so that the strategic rationale of the companies can be considered in addition to the analysis of the digital technology and business innovation. Several authors emphasise the importance of disruption and associate disruptive strategies with new entrants (Neale et al. 2020; Cosma and Rimo 2024). The logic is that new entrants have different strategic objectives, which centre around market disruption where they will have an advantage over incumbents and their legacy business models. However, the use of insurtech by incumbents is of equal importance and according to McKinsey in an article on the future of insurance, there is huge potential for incumbent competitors to exploit insurtech to defend their market positions (Balasubramanian et al. 2021a, b). Other research also identifies the importance of insurtech to improve existing business processes and products in existing business models of leading insurance firms (Neale et al. 2020). Innovation may therefore be in the form of a radical new business model by a new entrant, or improvements to existing processes and products by incumbents. To compare incumbent insurtech strategies with new entrants, a business innovation framework is developed in the next section.

## Business innovation

A logical method of relating insurtech to the application context of insurance markets is to focus on existing insurance processes in the form of a customer lifecycle approach (Balasubramanian et al. 2021a, b; EIOPA 2021), where each activity in the customer lifecycle is a business process, e.g. customer acquisition, risk assessment, pricing, claims payment and e-service. The first wave of AI applications and innovations can be mapped onto this model, e.g. see the work by EIOPA (2021). Product innovation is a key feature of most new entrants’ offers, e.g. flexible insurance based on mileage using GPS tracking, new risk mitigation in commercial insurance using smart sensors, or automated payments of claims based on an AI assessment of vehicle or property damage. Product and process innovation are established themes in the innovation literature (Utterback and Abernathy 1975) and the matrix framework of product and process innovation (Sosa and Montes 2022) brings together these dimensions to create some interesting insights that demonstrate their interdependency. Looking outwards from the firm there is a need to incorporate innovations in the value chain. Value chain innovation has been added to capture the effects of digital technologies that go beyond the organisational boundaries of a single firm and create new possibilities for the structure of market networks, which are an important source of innovation, e.g. smart business networks (Van Heck and Vervest 2007), integrated supply chains (Holland et al. 1992), electronic marketplaces (Bakos 1991) and new types of search intermediaries such as price comparison engines (Holland et al. 2016). The distribution of insurance is strategically important because of its role in marketing insurance services and managing the customer relationship.

In summary, the three types of innovations are: (1) the design, performance and management of insurance *business processes* (Snihur and Wiklund 2019); (2) the development and implementation of new insurance services from *product innovation* (Bucherer et al. 2012); and (3) innovation in the *value chain configuration* of insurance markets that is related to the customer journey concept from a customer perspective and the channel strategy of insurance companies (Catlin et al. 2017). The contribution of this framework is that it is a synthesis of innovation concepts from the general and insurance-specific literature, and it provides the basis for a systematic analysis and evaluation of innovation from insurtech in a variety of case vignette examples. To develop the theory in more detail, a sample of authoritative research into the three types of business innovation is presented in Table 1.

**Table 1:** Key references: insurtech business innovation framework

	Theoretical objectives	Analytical technique
Product innovation	Analyses the renewal of the firm from the interaction of firm competencies and product innovation through the separate processes of exploitation of existing resources and exploration of new firm capabilities (Danneels 2002)	Case study research and theoretical analysis of literature
	Impact of technology and demand heterogeneity on product and process innovation (Adner and Levinthal 2001)	Computer simulation
	Impact of organisational downsizing on product innovation (Dougherty and Bowman 1995)	Multiple case studies
	Product innovation in tandem with process improvements to enhance the overall business model in the context of dynamic capabilities and strategic leadership (Schoemaker et al. 2018)	Conceptual
	Continuous product innovation based on customer feedback and the proactive use of data for exploring new ideas, encouraging the development of new ideas and testing the effectiveness of product innovations (Werder et al. 2020)	Case study of a single organisation
	The role of digital technology in product innovation that identifies the importance of data platforms, distributed innovation and combinatorial innovation, which is a feature of digital technology (Yoo et al. 2012; Lyytinen et al. 2016)	Theoretical analysis and secondary data
Process innovation	Process innovation based on an open strategy approach to encourage further improvements from information sharing (Von Krogh et al. 2018)	Panel data and longitudinal case research
	Relationship between competitive strategy and innovation and the relationship between production process characteristics and innovation (Utterback and Abernathy 1975)	Systems dynamic model
	Impact of technology and demand heterogeneity on product and process innovation (Adner and Levinthal 2001)	Computer simulation
Value chain innovation	Smart business networks based on orchestration and flexible economic partnerships to encourage responsiveness in new product design and production flexibility(Van Heck and Vervest 2007)	Case studies of leading smart business networks
	Electronic marketplaces and their likely evolution based on information search theory and transaction cost economics (Bakos 1998)	Strategic analysis based on transaction cost economics
	Direct channel strategy that addresses the customer directly and disintermediates the traditional insurance broker channel in insurance (Channon 1998)	Case study of a single organisation and channel strategy theory
	Value creation in business-to-business relationships as part of a larger network (Kothandaraman and Wilson 2001)	Survey research of business organisations

### Product innovation

Examples of product innovation include behavioural insurance that utilise telematics in automotive insurance and fitness trackers in health insurance; parametric insurance that pays out automatically on the satisfaction of predefined rules or limits such as missed or late flights, movement in stock indices and currencies and extreme weather; usage-based insurance for gig-economy workers such as taxi drivers and Airbnb hosts; and novel insurance for new areas such as cyber insurance. Many of the innovations in insurance over the past 10 years have exploited a combination of digital technology (Internet of Things, telematics, sensors) and AI (machine learning, RPA, NLP, image recognition, predictive analytics, and data). Insurance is an information-based product, where the market is in the maturity stage of the product lifecycle. Therefore, non-technology improvements based on legacy business models tend to be incremental in nature and to build on existing products, processes and use traditional data sources. By observation, most of the current slew of innovations in insurance are made possible by new kinds of big data combined with AI technology and enabled by cheap, ubiquitous computing (Catlin et al. 2017). A focus on new forms of big data is therefore appropriate to explore the link between data and product innovation (Liu et al. 2018).

### Process innovation

Business processes are the set of activities involved in achieving a business goal or objective and produce an output such as service to an external customer or an internal organisational function. The key processes in an insurance firm based on practice from systems design are policy administration; product market development; underwriting; risk and compliance; customer management; and claims processing (IBM 2016). Innovation in business processes include increased automation, redesign and simplification of business processes and related legacy systems, novel techniques and processes to assess risk, new forms of e-service from apps and AI-powered call centres, and new methodologies for testing new product ideas. Process innovation may lead to or form a critical part of product innovation. For example, innovations in e-service processes form part of the overall product offer to customers or may make the insurance service easier to use thereby enhancing the product and the customer perceived value. Developments in analytics processes are also an integral component in behavioural-based insurance products.

### Value chain innovation

Value chain innovation in insurance is the reconfiguration of relationships throughout the insurance value chain, e.g. price comparison websites, data platforms and direct channel strategies (Eling and Lehmann 2018). Price comparison websites revolutionised the distribution of consumer insurance products, and more sophisticated AI comparison websites supported will enable more complex comparisons of product features, in addition to price. Two key attributes of price comparison tools are the ease of use and time-saving benefits that they offer to consumers, which are often overlooked because of the emphasis on lower prices.

The big data revolution is concerned with new types of data from a range of different sources: IoT, smart watches, sensors, telematics, smart buildings, and satellite imagery (EIOPA 2019). Initial research into big data focused on defining its key features, i.e. the 5Vs of volume, velocity, variety, veracity, and value (Chen et al. 2012), the analytics potential of big data (Gandomi and Haider 2015), and the importance of data granularity at scale. Looking beyond the technical attributes of big data (Catlin et al. 2018), the emergence of data platforms and related value chain networks (Eling and Lehmann 2018; Eling et al. 2021) will have a significant competitive impact on the insurance market because they will define the control over the collection, storage, access, use and evolution of data within and potentially across market sectors. McKinsey argues that platform business models consisting of buyers and sellers will account for 30% of revenue by 2025 (Catlin et al. 2018) and makes the important point that all the major technology companies, including Google and Amazon, operate platform business models. In markets such as Germany, where brokers still play a significant role in the distribution of insurance, a new kind of insurance company, US Disruptor, has launched and focused on two key areas: bypassing the broker to address the consumer directly and ease of use. The principal innovation here is channel strategy, where US Disruptor is using ecommerce and mobile technology to connect directly to individuals in the consumer market, in much the same way that Direct Line used the telephone and intensive television advertising in the 1980s (Channon 1998).

### Incumbent competitors versus new entrants

The competitive position of the insurance companies, incumbent or new entrant, is important because this difference in strategic perspective (Freeman and Engel 2007) is reflected in their insurtech and business innovation strategies (Christensen 1997). Incumbent firms typically focus on defending their market positions, e.g. by using digital technology to enhance existing business processes and products (Chesbrough and Appleyard 2007; Bogers et al. 2019) whereas new entrants often attempt to disrupt the status quo. The most ambitious type of new entrant is a firm such as US Disruptor (Barbera 2023) that enters as a new insurance company, i.e. it registers as an insurance company and is therefore subject to the insurance regulatory framework and must overcome marketing barriers to entry against large and established insurance companies. New entrants typically use insurtech to focus on a specific part of the insurance value chain such as risk modelling or distribution, and work in partnership with incumbent firms.

## Methodology and case data

The methodology is based on a case analysis of insurance firms using a combination of primary data for the incumbent, established competitors and secondary data for the new entrants (Eisenhardt and Graebner 2007).

### Sampling logic

The sampling strategy was based on the concept of theoretical sampling (Eisenhardt 1989) and influenced by the research objectives of exploring a new and rapidly changing technology and insurance markets. The theoretical sample is designed to capture a variety of innovations. The incumbent firms were chosen to represent one of business process, product and insurance value chain configuration. They are all international firms and each of them is a leader in AI and digital strategy, based on a combination of our own research with these firms and secondary data from industry conferences and business journals. The new entrants were part of a separate study where 88 UK insurtech companies were identified from a synthesis of data from Lloyd’s Lab (39), Crunchbase (17), new projects that were recently funded by government investments (12), disruptive lists and blogs (11) and industry presentations (9). All the insurtech firms were categorised based on a continuum from incremental change, re-engineering, strategic enhancement to disruptor (Neale et al. 2020). Of the 16 disruptors identified in the sample of 88 insurtech new entrants, four companies were chosen because they were established and therefore likely to be the most representative of future patterns of innovation and provided a variety of innovations in new product design. That is, these firms all have significant numbers of customers and can therefore demonstrate market credibility, scalability of the innovations that are described and the active use of innovative products. An overview of the theoretical case sample is shown in Table 2.

**Table 2:** Theoretical case sample based on insurtech innovation framework

Sample of insurance firms		Business innovation		
		**Business processes**	**Product**	**Insurance value chain configuration**
Incumbent insurance firms*	LEGACY CO	***Strategic enhancement and automation of legacy systems and processes***	Improvements in product performance, e.g. claims and e-service	Not applicable
	Price Comparison	Sophisticated customer journey and e-service processes	The price comparison tool for new customer acquisition and e-service emphasises user interface design for ease of use and speed of operation	***Value-chain disruptor through price comparison website***
	Behavioural Auto	Interactive communication with customers to improve driving performance	***Behavioural automotive insurance and new forms of big data***	Not applicable
New entrants**	Platform Insurance Services	Ease of use in customer-facing processes	***Product innovation based on personal insurance folder***	***Value-chain disruptor through data platform that connects a set of insurance firms directly with consumers through a data platform that is organised around the customer***
	PayAsYouGo	Risk mitigation through dynamic communication with customers	***Product innovation and use of behavioural data***	Partnership arrangement with incumbent for underwriting and direct marketing to consumers
	US Disruptor	Ease of use, speed and convenience based on AI chatbots for new customer acquisition and claims	***Product simplification based on an AI data flywheel and social giving are the defining characteristics of the product offer***	Value chain disruptor through direct channel strategy
	Flexi Cover	Focus on mobile platforms to simplify the customer acquisition process	***Product innovation is based on short-term and flexible cover, to disrupt the standard annual cycle for automotive insurance***	Direct marketing to a specific market segment through a mobile, digital first strategy

^*^This is an overview of detailed case studies that are based on primary data collection through detailed interviews with key stakeholders

^**^The analysis of new entrants is based on secondary data from industry conferences, company websites, business journals and news articles

Bold italics denotes high importance

The incumbent firms were chosen as exemplars to illustrate each type of business innovation. LEGACY CO is a market leader and is typical of an established, broad-based insurance firm. It is using AI technology to strengthen its existing business model and protect its market position, especially through automation of a wide range of internal and customer-facing business processes. Price Comparison.com is a market leader in price comparison websites, which have disrupted the insurance value chain and other market sectors such as energy and health, which makes it a multi-sided insurance platform (Hagiu and Wright 2015). Behavioural Auto is a leading consumer insurance company that has embraced telematics and behavioural insurance in the automotive sector, which uses risk-based pricing, based on driving performance.

The representativeness of the results and their wider validity for other insurance firms is an important issue and is considered separately for the incumbent firms and insurtech disruptors (Reynolds et al. 2003). The researchers were involved in a major UK-funded research project into insurtech and were therefore already closely involved with a range of incumbent firms and insurtech new entrants. The first point is that this is not meant to be a survey based on a random survey of many insurtech firms but rather a focused analysis of industry leaders, i.e. digital leaders as characterised by their use of AI and related digital technologies (Hall et al. 2024). The research objective of the paper is to explore the nature of innovation in digital leaders using the theoretical lens of incumbent firms versus new entrants. The innovation framework in Table 1 was used to select three incumbent firms that had established distinctive strategies for each of product, process and value chain configuration innovation. The size of the incumbent firms and their high market share means that they represent a significant part of their respective insurance markets. They were chosen because they were leaders—the focus of this research is to explore the use of advanced digital technologies and these firms are therefore likely to be representative of the *future* pattern of innovation in insurance markets. It cannot be claimed that they are representative of a current market because they were purposely chosen as digital leaders. However, notwithstanding the digital leader selection criterion, there is no reason to believe that they are unusual or atypical of leading insurance firms in Europe and the US.

The new entrants were part of a separate study of 88 insurtechs. The four insurtech firms were chosen from a sub-group of 16 ‘disruptors’ and were chosen because they were well established and therefore their strategies had already passed the validity acceptance test by many early adopters and early growth customer segments. In addition, these four insurtechs provided a variety of product innovations. In fact, a defining feature of the disruptor insurtechs is that they were all seeking to disrupt the insurance market with radically novel and innovative insurance products that exploited advanced digital technologies, supported by process and to a lesser extent value-chain innovations.

The geographic focus was on the UK market for the international firms LEGACY CO and Price Comparison, and for a large European market for Behavioural Auto. Although the data were collected in the UK only for LEGACY CO and Price Comparison, these firms are international with extensive interests throughout Europe and, in the case of LEGACY CO, also in the US insurance market. Behavioural Auto is a European-only insurance firm and the use of similar technology by other European firms suggests that these results are similar to other nationally focused automotive insurance companies in Europe. The insurtech firms were based in the UK except for US disruptor, which is headquartered in the US and has expanded into Europe with common offerings.

### Case study method

The case study research methodology was chosen because it is particularly well suited to exploring new and emerging phenomena that are not well understood and are in a state of flux. The concept of theoretical constructs for new theory building was used to collect, structure, and analyse data from multiple case study organisations (Volmar and Eisenhardt 2020; Eisenhardt and Graebner 2007). The advantages of case study research over other methodologies such as a survey, were that it allowed the researchers to analyse and evaluate the innovations in an organisational context and make sense of new and rapidly changing digital technology and business concepts. This intensive research methodology is much more likely to yield new and interesting insights than an extensive research design (Sayer 2010).

The case studies are in two groups: large incumbent firms and new entrants. This approach yielded theoretical diversity and illustrated the contrasting business innovation and insurtech strategies by incumbent firms with new entrants. The difference between incumbent firms and new entrants is a key distinction in analysing digital strategy (D’Ippolito et al. 2019; Volpentesta et al. 2023). However, the insurtech literature tends to focus on new entrants and neglects the insurtech strategies of incumbent firms.

The case data was collected and analysed as part of a major study of insurtech and innovation in insurance markets and considered both large incumbent firms and new entrants. The data set is comprised of primary data from three large, incumbent firms, and secondary data from four new entrants. The authors worked with each of the incumbent companies from 2018 to 2023. Interviews with key informants in senior management were supported by internal reports, documents, and external secondary data, which were used to add richness to the case analysis, and to corroborate interview data. The exploratory nature of the research was supported by semi-structured interview questionnaires that captured the strategic objectives of the innovation, the nature of the business innovation using the research framework model and an analysis of each individual AI application to explore how insurtech and innovation are related to each other, within a broader strategy context. This facilitated a flexible discussion of new ideas with practising managers who were leaders in innovation and insurtech. The new entrants all had high profiles at industry conferences and events, and promoted their innovations through advertising, industry journals and through their websites, which collectively provided a rich set of data that was used to evaluate their innovation focus, supported by digital technology and AI systems. The data was analysed based on the new entrants’ stated strategies, an analysis of innovations structured using our own research framework, and an analysis and evaluation of each individual insurtech example. An overview of the companies’ market positions and data collection strategy is given in Table 3. Tables 4 and 5 provide an analysis of the innovation strategies and associated use of digital technologies for the case sample.

**Table 3:** Overview of case sample and data collection strategy

		Overview	Data collection strategy
Incumbent insurance firms*	LEGACY CO	LEGACY CO is one of the top 5 European insurance companies measured by gross premiums in 2023	The data collection focused on the UK division. Interviews were conducted with 3 members of the team responsible for AI strategy and innovation. This was supported by secondary data in the insurance business press that described and outlined the AI strategy based on interviews with the CEO
	Price Comparison	Price Comparison is one of the market leaders in the UK, which is the most advanced market for car insurance price comparison in Europe. It has international offices in the EMEA region	The AI strategy was organised around individual projects that focused on process or product specific application areas, totally 8 main AI systems. Each project manager was interviewed, together with the IT director and the lead AI strategist. The interviews took place over a period of 3 years from 2018–2021 and were supported by detailed email exchanges and internal strategy documents
	Behavioural Auto	Behavioural Auto is a leading European car insurance company where it is the market leader in a large European country	Interviews were conducted with the head of data analytics and the behavioural insurance team and supported by internal presentations and written reports to provide statistical information about the marketing and performance of the behavioural insurance
New entrants**	Platform Insurance Services	UK insurtech company that has been established for over 20 years that focuses on ease of use through mobile platforms to connect and manage insurance services	The main sources of data were business presentations at industry conferences and supported by secondary data on case studies that were published in the industry press
	PayAsYouGo	A UK technology-driven insurtech that focuses on flexible automotive insurance based on usage rather than fixed time periods	This is an established company that has written order of magnitude (100 k) policies and has made several detailed presentations at industry and academic conferences, which analysed the evolution of its product-market and digital strategy, and its position within the broader insurance ecosystem. Business press articles also provided additional company data about its digital strategy and marketing performance
	US Disruptor	This is an insurance carrier and has launched in the US to focus on a specific market segment and is expanding into Europe	This company is a very high-profile insurance new entrant that has made tens of conference presentations and journal interviews. It has been the subject of detailed research by industry analysts and academics. A range of industry, academic and business conference presentations provided a wealth of secondary data
	Flexi Cover	Established insurtech that has insured over 1 million cars with flexible, short-term policies	The company promotes itself heavily through its own white papers about itself, industry presentations and has received widespread attention in the academic literature. These data sources provided a wealth of relevant data and concepts about its digital innovation strategy

**Table 4:** Incumbent firms’ case data

Theoretical construct/company	Market position and strategy	Insurtech, AI and big data	Business innovation	*Case discussion*
LEGACY CO	*LEGACY CO is a market leader in consumer and business markets that is using a variety of AI and digital technologies to defend its strong market position across a wide range of insurance lines*	*The principal data challenge it faces is to effectively manage the vast number and types of documents that are used in its systems, most of which contain unstructured information. LEGACY CO is implementing a range of AI applications: RPA in policy administration; simple NLP to extract and classify unstructured data; complex NLP for risk engineering and smart contract management; image recognition in claims management; and machine learning for fraud detection*	*The emphasis is on improving and augmenting the performance of the existing business model. Automation of existing processes is leading to significant productivity gains and cost savings. Analysis of unstructured data from NLP applications generates management insights from legacy systems and support the development of new risk engineering solutions and smart contract management capabilities*	*The focus of LEGACY CO’s AI strategy is to significantly improve its internal business processes, which creates administrative cost savings, reduction in the rate of fraudulent claims, and product enhancement through faster performance and easier to use services. The overall business model remains the same*
Price Comparison	*Price Comparison is a leading price comparison website in the UK and is part of an international group which has operations in the EMEA region. It entered the insurance market as a disruptor and has continued to adopt this mindset with its adoption and implementation of new technology, including AI, in many aspects of its business model*	*Price Comparison is implementing machine learning in several areas of its business to improve customer journey design and to provide highly relevant content to its customers. Two important aspects of the customer user experience that can be improved with AI are simplification of the customer journey and personalisation of information content*	*Price Comparison is using digital technology and AI to continually improve the effectiveness of its price comparison services that give consumers a fast and efficient method to compare products and prices from a wide range of providers. Customer journeys are evaluated for user experience and their effectiveness in providing the customer with an efficient and feature-rich experience*	*Price Comparison is a digital leader and focuses on the distribution segment of the value chain. It is using AI to improve customer-facing processes through a combination of simplifying the customer journey and personalising content to individual customers*
Behavioural Auto	*Behavioural Auto is a leading European home and automotive company. It is embracing new forms of big data to implement behavioural insurance in the automotive sector, which means that it is offering both traditional and behavioural automotive insurance in parallel*	*GPS, mobile phone data and potentially engine management data, weather data, and collective information from road authorities and the network of customers create a rich data set that is used to calculate an objective evaluation of a driver’s ability and expected safety, which is used to determine a risk-based and use-based pricing strategy*	*Behavioural insurance uses driving data instead of traditional data such as age, accident history and experience. Risk is treated as a dynamic variable and drivers are encouraged to improve their driving safety through active feedback that explains their driving score in a manner that can be understood by general, non-technical customers*	*Behavioural insurance creates expertise so that the company can respond quickly if the market grows. This blocking strategy impedes new entrants and improves the retention of existing customers. The business innovation stems from a new product, and business processes that capture, organise, and interpret new forms of big data. The innovation creates customer value in terms of flexibility, risk reduction and price advantages for careful drivers*

**Table 5:** New entrants’ case data

Theoretical construct/company	Market position and strategy	Insurtech, AI and big data	Business innovation	Case discussion
Platform Insurance Services	*Platform Insurance Services is an insurance platform that combines personal and business insurance products from a variety of insurance firms in a single app and offers a range of products including home, pet, car and commercial. The design of the app creates a smooth and fast user interface*	*Platform Insurance Services offers insurance *via* turnkey APIs, mobile app and web. The digital technology is characterised by smart user design and supported by a big data platform. Re-engineering of the buying process is a key feature of the offer*	*The business innovation is focused on a user interface that creates an easy-to-use online customer journey. The innovations are the search and buying processes, and an insurance data platform rather than the products themselves. The differentiation is the convenience and ease of use*	*Platform Insurance Services offers numerous insurance products *via* turnkey APIs, mobile and web app to simplify the customer experience. It has also started forming partnerships which enable insurers to cross-sell insurance*
PayAsYouGo	*PayAsYouGo offers a real-time pay-per-mile car insurance service. It is a disruptor, focusing on product innovation using new forms of big data from a vehicle’s engine management system and GPS driver data. It has partnered with LEGACY CO for the underwriting and uses its app to market directly to customers who value the flexibility of pay per journey*	*The PayAsYouGo app is used to acquire new customers and deliver the insurance service, with automated pricing information for each journey. The AI system is an algorithm that analyses the vehicle telematics information from the engine management system and GPS data to calculate route data, distance, and driving behaviour characteristics. Pricing and risk information are based on driving behaviour and historical loss data from the whole customer base*	*PayAsYouGo is a new entrant that offers a brand-new type of behavioural and pay-per-use car insurance service. The product innovation is underpinned by a range of business process innovations, including new customer acquisition, driver behaviour analytics, pricing and claims management*	*PayAsYouGo enjoys a first mover advantage in the UK for real-time pay-per-mile insurance. It focuses on product innovation and customer-facing processes and partners with LEGACY CO for back-office systems and underwriting. The natural curiosity of customers for new products helps its marketing and the partnership with LEGACY CO overcomes the regulatory hurdles for new entrants*
US Disruptor	*US Disruptor is an insurtech that deals directly with the customer. Its target market segment is young customers who value the use of technology to reduce the friction involved in buying insurance products. It applies AI throughout its business model, adopts an ethical position and shares its profits with charities*	*AI is used for: customer experience (CX); pricing and underwriting; and claims management. Virtual assistants provide quotes and handles payments. AI technology handles FNOL for 96% of claims and 1/3 of cases are managed with no human involvement. The AI bots operate independently and are supported by staff where necessary, e.g. in more complex cases*	*US Disruptor has re-engineered every aspect of the customer journey with automated AI-enabled ‘bots’ that can handle a significant proportion of customer queries, claims, and routine policy amendments. The AI systems and process-reengineering enable US Disruptor to address customers directly*	*A combination of product and channel innovation disrupts the market. Its strategy is a data-first perspective with an emphasis on world-class user experience. The pricing and underwriting capability was developed through the AI data flywheel effect and aggressive pricing. AI is used to refine and segment customers and for cross-selling*
Flexi Cover	*Flexi Cover is an innovator for customers that require short term car insurance. It is the UK’s first insurer to offer car insurance by the hour, which is marketed directly through mobile applications. All aspects of the motor insurance product lifecycle, i.e. search, buy and e-service, are managed through the mobile platform, with a 24/7 customer support system*	Via* Flexi Cover’s iOS and Android mobile app, customers can purchase short-term insurance within minutes. Flexi Cover's platform addresses the customer directly and does not require brokers or other search intermediaries, which reduces affinity marketing costs and makes what is initially a niche product economically viable*	*Time-based insurance service that covers very short periods of time ranging from a few hours to a few days is a key feature of insurance, e.g. temporary car insurance; learner insurance for a minimum of just one hour; and ‘pay as you go’ insurance*	*Flexi Cover has a first mover advantage in the UK with its flexible short-term insurance. Its focus on product innovation is complemented with an innovative interface, efficient product set up and sophisticated e-service. Insurance cover takes less than one minute to arrange*

## Case discussion

The case discussion is structured around cross-case analysis for each group, incumbent insurance firms and new entrants, followed by some general observations across the whole sample.

### Incumbent insurance firms

The incumbent firms are using insurtech and AI to defend market positions by improving their existing business models. A synthesis of the AI systems for incumbent firms is shown in Table 6.

**Table 6:** Incumbent firms: synthesis of AI systems and business innovation

AI systems	Business innovation
Automation of simple and repetitive tasks through RPA	Increased efficiency of routine business processes associated with legacy systems
Replacement of human operators in call centre operations through the advanced use of voice recognition, NLP and insurance ontology conversation frameworks	Process efficiency and product enhancement through faster customer services
Online chat in ecommerce systems using conversation bots that are trained on very large data sets	Process efficiency and enhanced customer experience
E-service through web browsers and mobile applications supported by chatbots	Process efficiency to reduce the cost to serve and improve availability and timeliness of e-service
Enhanced capabilities in risk analysis and pricing of new policies from the application of machine learning to model broad and large data sets that relate the characteristics of the insured asset to possible outcomes in terms of the frequency and magnitude of claims	Improved assessment of risk and fast, dynamic pricing for customers
Sophisticated market segmentation based on clustering analysis using machine learning techniques of diverse customer data, including online search behaviour, demographics, policy history, claims history, social network data and interactions between the insurer and the customer captured in CRM systems	Marketing process effectiveness from improved allocation of sales and advertising campaigns
Identification of fraudulent claims	Loss reduction from improved claims management
Automated regulatory compliance in both an active manner, i.e. to intervene in contentious decisions, and in reporting historical compliance	Administrative efficiency from better regulatory processes
Launch of behavioural insurance	Behavioural insurance product

The improvement of business processes generates simple efficiency benefits and improves process effectiveness, e.g. improved pricing from machine learning, fewer fraudulent claims, and better user experience. The incumbents’ market strategies are characterised as AI augmentation business models to defend strong market positions. Behavioural Auto’s behavioural insurance product blocks new entrants by making it more difficult for them to gain a foothold in the market and prepares the company for future market scenarios. Price Comparison is using AI for product innovation with an emphasis on user experience, e.g. improved customer service from automated search and e-service capabilities, speed and time savings for the customer, and ease of use. Its AI strategy is to enhance and augment its current business model.

LEGACY CO is using AI to improve all aspects of its existing business model, i.e. the focus is on strategic enhancement rather than radical change. It is doing this through a combination of automation of business processes, (Walker 2020), such as e-service, fraud detection and smart contract management. Improvements in business process performance also leads to improved product performance, e.g. faster customer service from improved e-service in areas such as claims and new business applications. LEGACY CO is also a research pioneer in areas such as predictive analytics and ethical use of AI systems.

New entrants that exploit AI and big data pose an existential threat to incumbents, but these examples of novel insurtech strategies by incumbent insurance firms demonstrate that the market leaders have been proactive in adopting insurtech in a defensive capacity to both improve their legacy systems and processes, and to include new product features that exploit AI capabilities. They have developed strategic alliances with AI start-ups that seek to partner with existing competitors, e.g. see the wide range of fintech companies that target insurance companies, as well as banks and e-commerce (Riikkinen et al. 2018). The partnerships between incumbents and AI start-ups have the potential to combine small company innovation and technology leadership with large company scale and distribution, which exploits their rich data resources and market knowledge that are vital in the training of new AI systems.

### New entrants

The new entrants have a broader range of business innovation, because they are focusing on how to exploit insurtech to disrupt insurance markets through radical innovation. Flexi Cover and PayAsYouGo are outstanding examples of the use of product innovation to create new forms of insurance with time flexibility and usage patterns that simply did not previously exist. All three types of value chain configuration are illustrated by the research sample: Price Comparison (price comparison website), Platform Insurance Services (data platform) and US Disruptor (direct channel strategy). These companies all create value through their interactions with customers. These high-profile new entrants all offer insurance services directly to customers and apply insurtech throughout the customer journey. Insurtech innovation often focuses on product innovation and new customer acquisition and these new entrants illustrate these characteristics. In addition, they also show the importance of very high-quality and easy to use e-service, because it facilitates better customer retention. Well-designed user-interfaces also reduce administrative costs and therefore make their business models more profitable.

Strong product innovation is a feature of the new entrants: Platform Insurance Services offers an easy-to-use bundled service that exploits a data platform; PayAsYouGo offers a pay-per-use behavioural insurance product; US Disruptor is positioned as an ethical insurance firm that shares its profits with customers’ charities; and Flexi Cover is focusing on product innovation by offering short-term insurance cover, which is largely neglected in the traditional annual-cycle automotive and home insurance markets. To establish a foothold in the insurance markets, the new entrants all have clear market segmentation strategies that focus on under-served segments, e.g. young car drivers, and customers who are early adopters of new technology and who value AI product innovations.

Platform Insurance Services’s data platform changes the way that providers offer their products through an intermediary in an integrated manner that enhances the customer experience and reduces the amount of time to manage the insurance documentation and policies. Platform Insurance Services uses the data platform to encourage affiliate marketing to increase its market reach. PayAsYouGo addresses its customers directly because its insurance service is not sufficiently standardised to be offered on a price comparison website, and it uses a partnership with an established insurance firm to manage the underwriting process and overcome the regulatory barriers to entry. US Disruptor is attacking markets where the retail brokers and intermediaries are strongest through intensive online marketing, coupled with a sophisticated interface that gives confidence to customers to buy directly without advice from an insurance broker. Flexi Cover also uses a hi-tech user interface to create a full self-service offering and have a direct relationship with customers. The AI systems and business innovations for new entrants are shown in Table 7.

**Table 7:** New entrants: synthesis of AI systems and business innovation

Insurtech and AI strategy	Business innovation
AI chatbot for new customer acquisition	Easy access to information and products and services to purchase
AI chatbot for claims management	Channel disruption with straightforward resolution of claims through automated insurance estimates and instant payment
AI chatbot for e-service requests	E-service process innovation that can also facilitate channel disruption by facilitating direct marketing of insurance
Behavioural driving risk assessment and pricing	Disruptive product innovation based on sophisticated data analytics
Pay-per-use insurance supported by AI to manage vehicle telematics and driver data	Product innovation through flexible short time-period insurance
Data platform for different types of insurance	Critical mass of customers and insurance providers in a single location, i.e. a data platform that acts as an electronic market
E-service to improve customer experience and reduce the cost to serve	Process innovation reducing cost to serve
AI market segmentation	Targeted marketing with tailored product offerings for each market segment

Bain & Company’s survey of 655 insurtech companies found that only 3% are insurance carriers. The bulk are technology firms that partner with incumbents and a relatively small number are creating new platforms and marketplaces. Partnerships between AI start-ups and incumbent firm accounts for most innovation. New entrants use AI for product innovation and engineer services specifically for digital channels. AI systems create new process capabilities, which are used to develop novel products and services, in conjunction with re-configuration of the insurance value chain.

### General observations

All the AI applications are examples of narrow AI because they address a specific, self-contained problem with a clearly defined objective. This is typical of AI in insurance applications and is in sharp contrast to the notion of general intelligence. US Disruptor’s leading-edge user interface that saves time and effort needed by customers to search, buy, and use the product. Platform Insurance Services focuses on user experience, and bundles insurance services from a range of suppliers on a single platform for convenience. The nature of the innovation is focused exclusively on user experience: ease of use; speed; convenience; and administrative efficiency, delivered through a mobile application. Behavioural insurance has the potential to disrupt health, buildings, cyber and automotive insurance and requires new forms of big data. Big data and data platforms are likely to change the competitive landscape of insurance, leading to new forms of insurance ecosystems and strategic partnerships (Ngai 2018).

## Conclusions

This research set out to explore the nature of AI strategy and insurtech business innovation from the strategic perspectives of new entrants and established competitors, which was a fruitful approach because the innovations in these two groups demonstrate that strategy context matters. Established competitors have different strategic priorities and natural advantages compared with new entrants, which influence and partly determine their AI strategies and more generally, the deployment of insurtech. Their market share gives them access to high volumes of data concerning existing insurance services (Keller et al. 2018), which gives them an advantage in developing AI to improve existing business models in the areas of customer service, underwriting, claims management and marketing. These ‘narrow’ AI applications naturally map onto a customer lifecycle model, for example see EIOPA (2019). An important consideration for incumbents is to reduce the cost to serve customers to improve profit margins and this is reflected in investments into business process improvements, especially those that enhance customer service levels whilst reducing costs, e.g. through smart chatbots and increased automation of online quotes, claims management and renewal processes. Incumbent firms are also actively improving the digital interface with their customers, and this is typically done within a legacy technology context, which makes it more difficult than starting from a clean slate.

Behavioural Auto is unusual as an established competitor because it was chosen specifically for its prowess in exploiting a market discontinuity that arose because of the availability of GPS technology and smart mobile phones that generate big data to dynamically evaluate driver risk. It should be noted though that this was done in parallel with their historical products, and still represents a relatively small but growing part of their business. An important element of insurtech strategy for all of the incumbent firms was to develop partnership strategies with small insurtech companies in addition to their large digital partners so that they could gain access to new and cutting-edge ideas and use them to strategically enhance their existing business models.

Conversely, new entrants must develop technology and concepts that have a significant advantage over existing approaches and legacy systems to build realistic marketing opportunities, often through partnerships with existing insurance companies. The natural starting point is product innovation to disrupt the status quo, because this is the area where their technology has the biggest impact. The other major area of innovation for new entrants is the reconfiguration of the value chain, e.g. see the direct channel strategy of US Disruptor, and the data platform concept offered by Platform Insurance Services. Although it is extremely difficult to enter the market as a new insurance carrier, because of market entry barriers from capital and regulatory requirements, US Disruptor has taken this approach and developed a full technology infrastructure from scratch, where it engineered all its services and designed them specifically for the digital channel. Its major weakness was its lack of underwriting experience and historical loss data, which meant that it incurred significant claims losses before it started to improve risk analysis as it developed its own claims and loss database.

The business innovation research framework is a synthesis of existing ideas in the literature but its application to a set of case studies in insurance is a novel one and therefore contributes to the literature by applying an adapted framework in a new context, and with a new set of empirical data. It is the basis for a new, dynamic, capabilities model of innovation in insurance. The innovation process can be simplified into the following proposed model: insurtech, AI and big data create new process capabilities in narrow business activities such as pricing, risk assessment and claims management. Processes with enhanced capabilities support and facilitate the development of new products and enable new value chain configuration patterns. The user interface is an important area of differentiation because it determines how consumers search, buy and use insurance products and is therefore important for both new entrants *and* incumbent insurance firms.

Insurance is inherently an information product, which means that the digital transformation of insurance markets is inevitable—the research questions and managerial problems are therefore concerned with how will insurtech transform markets, and what are the likely emerging patterns of business innovation? We have shown empirically that AI and big data are central components of insurtech and play a crucial role in many of the business innovation strategies. All the companies in the sample enjoy excellent access to the AI technology itself—the competitive battle is about access to relevant big data, especially risk and loss data to inform underwriting and pricing decisions. The shift towards behavioural insurance is therefore an opportunity for new entrants because historical insurance information is much less valuable where new forms of big data and AI are required. The cross-case analysis reveals that all the insurtech and AI systems were applied in narrowly defined insurance business processes. This empirical observation is important because it indicates that AI is at an early stage of maturity. These AI silos are likely to develop into organisation-wide systems, similar to the historical development and evolution of integrated enterprise resource planning systems (Holland and Light 1999).

The research sample was purposely designed to represent digital leaders because the research was focused on exploring the nature of innovation from advanced insurtech and there would have been relatively few insights from looking at companies that were only average or digital laggards. In addition, the leaders have already demonstrated the validity of these new innovations because significant numbers of customers have adopted them. The incumbent sample are also high market share firms and therefore represent a significant part of their respective insurance markets. It could therefore be argued that LEGACY CO is broadly representative of the likely evolution of insurtech innovation for legacy insurance carriers, and it is deploying these ideas in all its European and US markets. In price comparison, the UK is at the vanguard of new developments and Price Comparison therefore gives a good picture of emerging trends and innovation patterns in Europe. The exception in the incumbent group is Behavioural Auto, which stands out amongst incumbent firms because it has embraced such a radical new business model, albeit it in parallel with existing, historical products.

Some brief observations on the broader policy and societal implications of the research are that insurtech originates from both small companies and established insurance firms, and industry bodies and governments seeking to encourage innovation should actively promote AI applications in new entrants and incumbent insurance firms. Insurance is a vital part of all societies because it protects individuals and businesses from the consequences of risk and unforeseen events and innovation plays a crucial role in making insurance services more affordable, and effective. Some of the intractable problems in insurance such as exclusion may be at least partly solved by the novel use of digital technology so there is a clear incentive to encourage and facilitate insurtech through industry bodies, regulators and government funding for R&D.

Some promising areas for future research are outlined. To capture the diversity of business models and patterns of innovation in insurance, different theoretical lenses, e.g. innovation, business model, ecosystem and technology-focused theories provide complementary insights into the success and value of insurtech innovation. The concepts of data platforms and ecosystems are likely to grow in importance, especially if the data platforms are owned and managed by non-insurance firms such as automotive, technology, construction and health companies. The conclusions and proposed models suggested by this research could be used to inform a broader survey style approach to test their generalisability across a larger sample. Another exciting avenue of research is to conduct detailed, longitudinal studies of individual organisations that address the implementation challenges and new opportunities arising from insurtech and business innovation.

## References

[CR1] Adner, R., and D. Levinthal. 2001. Demand heterogeneity and technology evolution: Implications for product and process innovation. *Management Science* 47 (5): 611–28. 10.1287/mnsc.47.5.611.10482.

[CR2] Bakos, J.Y. 1991. A strategic analysis of electronic marketplaces. *MIS Quarterly: Management Information Systems* 15 (3): 295–310. 10.2307/249641.

[CR3] Bakos, Y. 1998. “The Emerging Role of Electronic Marketplaces on the Internet.” *Communications of the ACM* 41 (8). Association for Computing Machinery (ACM): 35–42. 10.1145/280324.280330.

[CR4] Balasubramanian, R., A. Libarikian, and D. McElhaney. 2021a. “Insurance 2030—The Impact of AI on the Future of Insurance.” *Mckinsey.Com*. https://www.mckinsey.com/industries/financial-services/our-insights/insurance-2030-the-impact-of-ai-on-the-future-of-insurance#/.

[CR5] Balasubramanian, R., A. Libarikian, and D. McElhaney. 2021b. “Insurance 2030-The Impact of AI on the Future of Insurance.” https://www.mckinsey.com/industries/financial-services/our-insights/insurance-2030-the-impact-of-ai-on-the-future-of-insurance.

[CR6] Barbera, S.L. 2023. Insurtech revolution in the insurance sector: A comprehensive review of the transformational impact and the lemonade case study. *International Journal of Management Research and Economics* 3 (2): 57–64. 10.51483/IJMRE.3.2.2023.57-64.

[CR7] Behm, S., U. Deetjen, S. Kaniyar, N. Methner, and B. Münstermann. 2019. “Digital Ecosystems for Insurers : Opportunities through the Internet of Things.” *Insurance Practice*, no. January: 1–10.

[CR8] Bogers, M., H. Chesbrough, S. Heaton, and D.J. Teece. 2019. Strategic Management of Open Innovation: A Dynamic Capabilities Perspective. *California Management Review* 62 (1): 77–94. 10.1177/0008125619885150.

[CR9] Bucherer, E., U. Eisert, and O. Gassmann. 2012. Towards systematic business model innovation: Lessons from product innovation management. *Creativity and Innovation Management* 21 (2): 183–198. 10.1111/j.1467-8691.2012.00637.x.

[CR10] Buesnel, C., N. Naumann, K. Mitzner, P. Evans, W. Wilson, and G. Narula. 2020. “This Time Is Different Six Trends That Will Determine the Future of Global Non-Life Reinsurance.” https://www2.deloitte.com/content/dam/Deloitte/ch/Documents/financial-services/deloitte-ch-en-fs-future-of-reinsurance-report.pdf.

[CR11] Catlin, T., J.-T. Lorenz, B. Münstermann, P. Braad Olesen, and V. Ricciardi. 2017. “Insurtech — the Threat That Inspires.” *McKinsey & Company*. www.mckinsey.com/clientservice/financial_services.

[CR12] Catlin, T., J.-T. Lorenz, J. Nandan, S. Sharma, and A. Waschto. 2018. “Insurance beyond Digital: The Rise of Ecosystems and Platforms.” *The-Digital-Insurer.Com*.

[CR13] Channon, D.F. 1998. The strategic impact of IT on the retail financial services industry. *Journal of Strategic Information Systems* 7 (3): 183–97. 10.1016/S0963-8687(98)00027-4.

[CR14] Chen, H., R.H.L. Chiang, and V.C. Storey. 2012. “Business Intelligence and Analytics: From Big Data to Big Impact.” *MIS Quarterly: Management Information Systems* 36 (4). University of Minnesota: 1165–88. 10.2307/41703503.

[CR15] Chesbrough, H.W., and M.M. Appleyard. 2007. *Open Innovation and Strategy*. California: University of California Press. 10.2307/41166416.

[CR16] Christensen, C.M. 1997. *The Innovator’s Dilemma: When New Technologies Cause Great Firms to Fail. The Innovator’s Dilemma: When New Technologies Cause Great Firms to Fail*. Boston: Harvard Business School Press.

[CR17] Cosma, S., and G. Rimo. 2024. Redefining insurance through technology: achievements and perspectives in insurtech. *Research in International Business and Finance*. 10.1016/J.RIBAF.2024.102301.

[CR18] D’Ippolito, B., A. Messeni Petruzzelli, and U. Panniello. 2019. Archetypes of incumbents’ strategic responses to digital innovation. *Journal of Intellectual Capital*. 10.1108/JIC-04-2019-0065/FULL/PDF.

[CR19] Danneels, E. 2002. The dynamics of product innovation and firm competences. *Strategic Management Journal* 23 (12): 1095–1121. 10.1002/smj.275.

[CR20] Dougherty, D., and E.H. Bowman. 1995. The effects of organizational downsizing on product innovation. *California Management Review* 37 (4): 28–44. 10.2307/41165809.

[CR21] EIOPA. 2019. “Big Data Analytics in Motor and Health Insurance.” Frankfurt. https://register.eiopa.europa.eu/Publications/EIOPA_BigDataAnalytics_ThematicReview_April2019.pdf.

[CR22] EIOPA. 2021. “Artificial Intelligence Governance Principles: Towards Ethical and Trustworthy Artificial Intelligence in the European Insurance Sector: A Report from EIOPA’s Consultative Expert Group on Digital Ethics in Insurance.”

[CR23] Eisenhardt, K.M. 1989. Building theories from case study research. *Academy of Management Review* 14 (4): 532–550. 10.5465/amr.1989.4308385.

[CR24] Eisenhardt, K.M., and M.E. Graebner. 2007. Theory building from cases: Opportunities and challenges. *Academy of Management Journal*. 10.5465/AMJ.2007.24160888.

[CR25] Eling, M., and M. Lehmann. 2018. “The Impact of Digitalization on the Insurance Value Chain and the Insurability of Risks.” *Geneva Papers on Risk and Insurance: Issues and Practice* 43 (3). Palgrave Macmillan Ltd.: 359–96. 10.1057/s41288-017-0073-0.

[CR26] Eling, M., D. Nuessle, and J. Staubli. 2021. “The Impact of Artificial Intelligence along the Insurance Value Chain and on the Insurability of Risks.” *Geneva Papers on Risk and Insurance: Issues and Practice*. Palgrave Macmillan. 10.1057/s41288-020-00201-7.

[CR27] European Commission. 2020. “White Paper: On Artificial Intelligence - A European Approach to Excellence and Trust.”

[CR28] Freeman, J., and J.S. Engel. 2007. Models of Innovation: Startups and mature corporations. *California Management Review*. 10.2307/41166418.

[CR29] Gandomi, A., and M. Haider. 2015. Beyond the Hype: Big Data Concepts, Methods, and Analytics. *International Journal of Information Management* 35 (2): 137–144. 10.1016/j.ijinfomgt.2014.10.007.

[CR30] Gorzata Ś.M., Mal, A. Koshiyama, and P. Treleaven. 2021. “Algorithms in Future Insurance Markets.” *Int.J.Data.Sci. and Big Data Anal,* 1 (1): 1–19. 10.51483/IJDSBDA.1.1.2021.1-19.

[CR31] Hagiu, A., and J. Wright. 2015. Multi-sided platforms. *International Journal of Industrial Organization*. 10.1016/j.ijindorg.2015.03.003.

[CR32] Hall, B., E. Lamarre, R. Levin, J.-T. Lorenz, and P. Simon. 2024. “Rewired and Running Ahead: Digital and AI Leaders Are Leaving the Rest Behind.” *McKinsey Digital*. https://www.mckinsey.com/capabilities/mckinsey-digital/our-insights/rewired-and-running-ahead-digital-and-ai-leaders-are-leaving-the-rest-behind.

[CR33] Heck, E.V., and P. Vervest. 2007. Smart business networks: How the network wins. *Communications of the ACM* 50 (6): 28–37. 10.1145/1247001.1247002.

[CR34] Holland, C.P., and B. Light. 1999. Critical success factors model for ERP implementation. *IEEE Software*. 10.1109/52765784.

[CR35] Holland, C., G. Lockett, and I. Blackman. 1992. Planning for electronic data interchange. *Strategic Management Journal*. 10.1002/smj.4250130706.

[CR36] Holland, C.P., J.A. Jacobs, and S. Klein. 2016. The role and impact of comparison websites on the consumer search process in the US and german airline markets. *Information Technology and Tourism* 16 (1): 127–148. 10.1007/s40558-015-0037-9.

[CR37] Holland, C.P., and A.S. Kavuri. 2021. “Artificial Intelligence and Digital Transformation of Insurance Markets.” *Journal of Financial Transformation* 54 (November). https://www.capco.com/Capco-Institute/Journal-54-Insurance/Artificial-Intelligence-And-Digital-Transformation-In-Insurance-Markets.

[CR38] IBM. 2016. “IBM Insurance Process and Service Models, General Information Manual.” https://www.ibm.com/downloads/cas/VDZDBOGP.

[CR39] Keller, B., M. Eling, H. Schmeiser, M. Christen, and M. Loi. 2018. “Big Data and Insurance: Implications for Innovation, Competition and Privacy.” https://www.genevaassociation.org/sites/default/files/research-topics-document-type/pdf_public/big_data_and_insurance_-_implications_for_innovation_competition_and_privacy.pdf.

[CR40] Kothandaraman, P., and D.T. Wilson. 2001. The future of competition: Value-creating networks. *Industrial Marketing Management* 30 (4): 379–389.

[CR41] Krogh, G.V., T. Netland, and M. Wörter. 2018. Winning with open process innovation. *MIT Sloan Management Review* 59 (2): 53–56.

[CR42] Liu, Y., J. Peng, Z. Yu - Proceedings of the 2018 International Conference, and undefined 2018. 2018. “Example.” *Dl.Acm.Org*, August. Association for Computing Machinery, 31–35. 10.1145/3297730.3297743.

[CR43] Lyytinen, K., Y. Yoo, and R.J. Boland. 2016. Digital product innovation within four classes of innovation networks. *Information Systems Journal*. 10.1111/isj.12093.

[CR44] Naylor, M. 2017. *Insurance Transformed: Technological Disruption*.

[CR45] Neale, F.R., P. Peterson Drake, and T. Konstantopoulos. 2020. “InsurTech and the Disruption of the Insurance Industry.” *Journal of Insurance Issues* 43 (2): 64–96. https://www.jstor.org/stable/26931211.

[CR46] Ngai, Joe. 2018. “Building a Tech-Enabled Ecosystem: An Interview with Ping An’s Jessica Tan.” *McKinsey Quarterly*.

[CR47] Reynolds, N.L., A.C. Simintiras, and A. Diamantopoulos. 2003. Theoretical justification of sampling choices in international marketing research: Key issues and guidelines for researchers. *Journal of International Business Studies*. 10.1057/PALGRAVE.JIBS.8400000/METRICS.

[CR48] Riikkinen, M., H. Saarijärvi, P. Sarlin, and I. Lähteenmäki. 2018. Using artificial intelligence to create value in insurance. *International Journal of Bank Marketing*. 10.1108/IJBM-01-2017-0015.

[CR49] Santenac, I., E. Majkowski, P. Vermeulen, and A. Sun-Young Bong. 2024. “2024 Global Insurance Outlook: Strengthening Trust to Unlock Innovation and Growth.” Accessed October 17. https://assets.ey.com/content/dam/ey-sites/ey-com/en_gl/topics/insurance/insurance-pdfs/ey-2024-global-insurance-outlook-report-v2.pdf.

[CR50] Sayer, A. 2010. “Method in Social Science: A Realist Approach: Revised Second Edition.” *Method in Social Science: A Realist Approach: Revised Second Edition*, March. Routledge Taylor & Francis Group, 1–213. 10.4324/9780203850374/METHOD-SOCIAL-SCIENCE-ANDREW-SAYER

[CR51] Schoemaker, P.J.H., S. Heaton, and David Teece. 2018. Special section on VUCA innovation, dynamic capabilities, and leadership. *California Management Review* 61 (1): 15–42. 10.1177/0008125618790246.

[CR52] Singh, S.K., and M. Chivukula. 2020. A Commentary on the Application of Artificial Intelligence in the Insurance Industry. *Trends Artif Intell* 4 (1): 75–79. 10.36959/643/305.

[CR53] Snihur, Y., and J. Wiklund. 2019. Searching for innovation: Product, process, and business model innovations and search behavior in established firms. *Long Range Planning*. 10.1016/j.lrp.2018.05.003.

[CR54] Sosa Gómez, I., and Ó. Montes Pineda. 2023. What is an insurtech? A scientific approach for defining the term. *Risk Management and Insurance Review*. 10.1111/RMIR.12243.

[CR55] Sosa, I., and Ó. Montes. 2022. Understanding the Insurtech dynamics in the transformation of the insurance sector. *Risk Management and Insurance Review*. 10.1111/RMIR.12203.

[CR56] Trajtenberg, M. 2019. Artificial Intelligence as the Next GPT: A Political-Economy Perspective. In *The Economics of Artificial Intelligence, An Agenda*, ed. Ajay Agrawal, Joshua Gans, and Avi Goldfarb. Chicago: The University of Chicago Press.

[CR57] Utterback, J.M., and W.J. Abernathy. 1975. A dynamic model of process and product innovation. *Omega* 3 (6): 639–656. 10.1016/0305-0483(75)90068-7.

[CR58] Volmar, E., and K.M. Eisenhardt. 2020. Case study research: A state-of-the-art perspective. In *Business and management, Oxford research encyclopedias*, 2020th ed., ed. E. Volmar and K.M. Eisenhardt. Oxford: Oxford University Press. 10.1093/acrefore/9780190224851.013.206.

[CR59] Volpentesta, T., E. Spahiu, and P. De Giovanni. 2023. A survey on incumbent digital transformation: A paradoxical perspective and research agenda. *European Journal of Innovation Management*. 10.1108/EJIM-01-2023-0081/FULL/PDF.

[CR60] Walker, A. 2020. “AXA Pilots AI-Powered Claims Tool, To Speed FNOL Process – Insurance Edge.” *Insurance Edge*. https://insurance-edge.net/2020/02/12/axa-pilots-ai-powered-claims-tool-to-speed-fnol-process/.

[CR61] Werder, K., S. Seidel, J. Recker, N. Berente, J. Gibbs, N. Abboud, and Y. Benzeghadi. 2020. Data-driven, data-informed, data-augmented: How ubisoft’s ghost recon wildlands live unit uses data for continuous product innovation. *California Management Review*. 10.1177/0008125620915290.

[CR62] Yoo, Y., R.J. Boland, K. Lyytinen, and A. Majchrzak. 2012. Organizing for Innovation in the Digitized World. *Organization Science* 23 (5): 1398–1408. 10.1287/orsc.1120.0771.

[CR63] Zanke, P., and D. Sontakke. 2021. “Artificial Intelligence Applications in Predictive Underwriting for Commercial Lines Insurance.” *Advances in Deep Learning Techniques* 1 (1): 23–38. https://thesciencebrigade.com/adlt/article/view/181.

